# Integrated stress response inhibition provides sex-dependent protection against noise-induced cochlear synaptopathy

**DOI:** 10.1038/s41598-020-75058-w

**Published:** 2020-10-22

**Authors:** Stephanie L. Rouse, Ian R. Matthews, Jiang Li, Elliott H. Sherr, Dylan K. Chan

**Affiliations:** 1 Department of Otolaryngology-Head and Neck Surgery, University of California, San Francisco (UCSF), 513 Parnassus Ave, Rm 719, San Francisco, CA 94143 USA; 2 Department of Neurology, UCSF, 675 Nelson Rising Lane, Room 214B, San Francisco, CA 94158 USA; 3 Department of Pediatrics, Institute of Human Genetics, Weill Institute for Neurosciences, UCSF, San Francisco, USA

**Keywords:** Cochlea, Inner ear

## Abstract

Noise-induced hearing loss (NIHL) is a common health concern with significant social, psychological, and cognitive implications. Moderate levels of acoustic overstimulation associated with tinnitus and impaired speech perception cause cochlear synaptopathy, characterized physiologically by reduction in wave I of the suprathreshold auditory brainstem response (ABR) and reduced number of synapses between sensory hair cells and auditory neurons. The unfolded protein response (UPR), an endoplasmic reticulum stress response pathway, has been implicated in the pathogenesis and treatment of NIHL as well as neurodegeneration and synaptic damage in the brain. In this study, we used the small molecule UPR modulator Integrated Stress Response InhiBitor (ISRIB) to treat noise-induced cochlear synaptopathy in a mouse model. Mice pretreated with ISRIB prior to noise-exposure were protected against noise-induced synapse loss. Male, but not female, mice also exhibited ISRIB-mediated protection against noise-induced suprathreshold ABR wave-I amplitude reduction. Female mice had higher baseline wave-I amplitudes but greater sensitivity to noise-induced wave-I reduction. Our results suggest that the UPR is implicated in noise-induced cochlear synaptopathy, and can be targeted for treatment.

## Introduction

Noise induced hearing loss (NIHL) affects nearly 20% of adults, with significant impact on social, psychological and cognitive function. In cochlear synaptopathy, moderate noise exposure that does not cause permanent shifts in hearing thresholds can nonetheless result in permanent loss of synapses between cochlear inner hair cells (IHCs) and spiral ganglion neurons (SGNs), associated with permanent reduced amplitude of wave I of the suprathreshold auditory brainstem response (ABR)^1^. These anatomic and physiologic findings are thought to correlate behaviorally with tinnitus and impaired speech perception in complex acoustic environments, both hallmarks of human auditory functional disability^2^.

The mechanism of synaptic damage in response to noise is incompletely understood. Glutamate excitotoxicity—synaptic damage stemming from increased glutamate release from overstimulated IHCs—has been proposed as a primary cause of noise-induced cochlear synaptopathy^3,4^. Recent work by our group has implicated the unfolded protein response (UPR) pathway in permanent NIHL. The UPR is a series of highly conserved processes related to endoplasmic reticulum (ER) stress that regulate protein folding and processing, and mediate downstream homeostatic and apoptotic responses. Multiple small-molecule modulators of the UPR have been developed, including Integrated Stress Response InhiBitor (ISRIB), a drug that activates eIF2B, downregulates ATF4, and leads to decreased synthesis of the pro-apoptotic UPR protein CHOP (C/EBP-homologous protein)^5^. We found that 106 dB SPL noise exposure, which produces permanent shifts in hearing thresholds, induces CHOP expression, and that ISRIB treatment provides partial protection against hearing loss and hair-cell death in this model^6^. These results warranted further study of the protective effect of ISRIB at lower levels of noise exposure, such as those involved in cochlear synaptopathy.

Further rationale for this approach is provided by a growing body of evidence implicating the UPR and ER stress in synaptic plasticity and neurodegeneration. UPR upregulation has been found in brains of multiple human and mouse models of neurodegeneration^7,8^. UPR dysregulation results in broad disruption of protein production, triggering neuronal death as well as disruption of calcium signaling, essential for maintenance of neurons. Manipulation of the UPR protects neurons in multiple neurodegenerative disease models; as such, it has been an appealing target of study for potential treatment of neurodegeneration^9^. The UPR also appears to be involved in the synthesis of proteins involved in synaptic function and structural maintenance. It is a major driver of synaptic loss through reduction in global protein synthesis rates, leading to reduction in synaptic plasticity in several neurologic diseases. Specifically, downregulation of ATF4 has been shown to promote neuroprotection, synaptic plasticity, long-term potentiation, and memory formation, suggesting that ISRIB would be effective in treatment of models of synaptic dysfunction in the central nervous system^10,11^. Indeed, ISRIB has been shown to rescue synaptic plasticity in a mouse model of Down syndrome, restore cell-specific synaptic function in a mouse model of repetitive traumatic brain injury, reverse trauma-induced hippocampal-dependent learning and memory deficits, and improve memory in wild-type and memory-impaired mice^5,12–14^.

The involvement of the UPR and efficacy of ISRIB in both neurodegeneration and synaptic maintenance in the brain led us to hypothesize similar roles for the UPR and ISRIB in the cochlea. In this study, we extend our finding that ISRIB can prevent NIHL^4^ and demonstrate that ISRIB treatment also protects against noise-induced cochlear synaptopathy.

## Results

### Female CBA/J mice have higher baseline ABR wave-I amplitude than male mice

**Figure 1:**
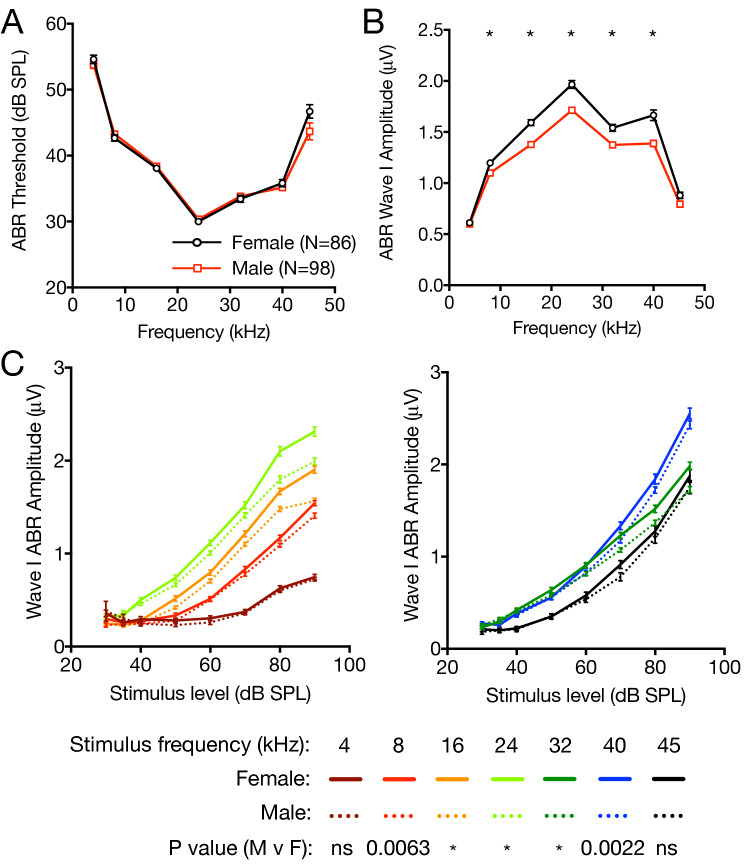
Baseline sex differences in ABR threshold and wave-I amplitude. 7 to 8-week-old female (black, N = 86) and male (red, N = 98) CBA/J mice underwent Auditory Brainstem Response (ABR) recording. ABR thresholds (**A**), average wave-I peak amplitudes (**B**) in response to suprathreshold tone pips at 70, 80 and 90 dB SPL, and growth of wave-I amplitude with stimulus presentation level (**C**) were measured. (**A**,**B**) Mean thresholds and wave-I amplitudes were compared between male and female mice at each frequency using Student’s unpaired two-tailed *t* test with Bonferroni’s correction for multiple comparisons at 7 frequencies. (**C**) ABR wave-I growth functions were compared in female (solid lines) and male (dotted lines) mice at individual frequencies (low frequencies, left; and high frequencies, right) by 2-way ANOVA by sex, with ABR stimulus presentation level as a repeated measure. *p* values are presented to demonstrate statistical differences by sex. Data represent means ± s.e. *p < 0.001.  *ns* not significant.

The physiologic manifestation of cochlear synaptopathy is reduction in the amplitude of wave I of the suprathreshold ABR, which serves as an indicator of the function of SGNs stimulated by ribbon synapses onto IHCs^15^. Because sex differences in ABR wave-I amplitude have been observed in humans, we tested both male and female mice and accounted for sex differences throughout all auditory physiology experiments^16^. To investigate baseline differences in wave-I amplitude, we measured ABRs on 86 female and 98 male CBA/J mice between 7 and 8 weeks old. While there was no difference in ABR thresholds between male and female mice (Fig. 1A), growth of wave-I amplitude with increasing stimulus presentation level and average suprathreshold (in response to 70–90 dB SPL stimuli) wave-I peak amplitudes were significantly higher in female mice between 8 and 40 kHz (Fig. 1B,C).

### Noise-induced ABR threshold and wave-I amplitude shifts differ in male and female mice

**Figure 2:**
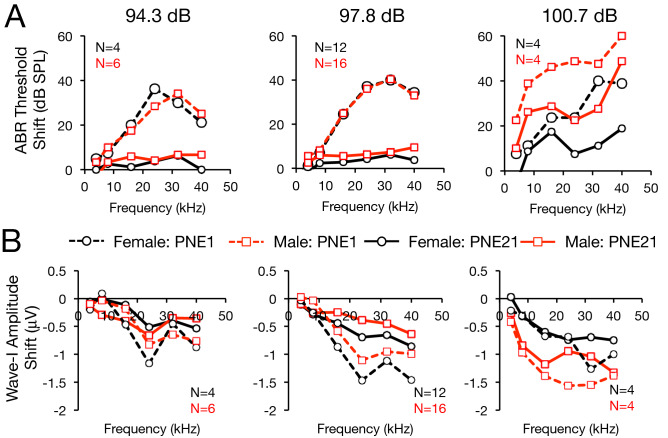
Sex differences in ABR threshold and suprathreshold wave-I amplitude shift after different levels of noise exposure. 8-week-old female (black) and male (red) CBA/J mice were exposed to 2 h, 8–16 kHz octave-band noise at the indicated sound pressure levels. ABRs were measured at baseline, 1 (dashed lines), and 21 (solid lines) days post noise exposure (PNE). Shifts in ABR thresholds (**A**) and suprathreshold wave-I amplitudes (**B**) relative to baseline were measured. 3-way ANOVA for sex, frequency, and noise exposure level indicated significant main effects of each factor on ABR threshold shift at PNE1, and on both threshold and wave-I shifts at PNE21, and interaction between sex and noise exposure level (*p* < 0.05 for all comparisons). Subgroup analysis at each noise-exposure level with 2-way ANOVA for sex and frequency revealed significant effect of sex at PNE21 on ABR threshold at 97.8 dB (*p* = 0.02) and 100.7 dB (*p* = 0.005), but not at 94.3, and on wave-I amplitude at 97.8 dB only (*p* = 0.02).

Having established these baseline sex differences, we sought to determine the efficacy of ISRIB to prevent wave-I amplitude reduction across multiple noise-exposure levels in a sex selective manner. First, to precisely define our cochlear synaptopathy model, we exposed male and female mice to a range of sound pressure levels. 97.8 dB SPL 8–16-kHz octave-band noise produced immediate elevation in ABR thresholds at PNE1 that then returned to baseline by PNE21, accompanied by large permanent wave-I amplitude shifts (Fig. 2A,B, middle). Lower noise-exposure levels produced similar outcomes—minimal threshold shift with more modest wave-I amplitude shifts (Fig. 2A,B, left, for 94.3 dB SPL). Even slightly higher noise-exposure levels, however, gave rise to substantial permanent threshold shifts (Fig. 2A,B, right, for 100.7 dB SPL; similar findings for 99.0 and 101.4 dB SPL, data not shown). These findings led us to use only the 94.3 and 97.8-dB levels as a model in subsequent experiments for noise-induced cochlear synaptopathy without threshold shift. Significant sex differences were observed in threshold shift and wave-I amplitude shift after 97.8 dB noise exposure, leading us to account for sex in all subsequent analyses.

### Noise-induced ABR threshold and wave-I amplitude shifts are mitigated by ISRIB in male, but not female, mice

**Figure 3:**
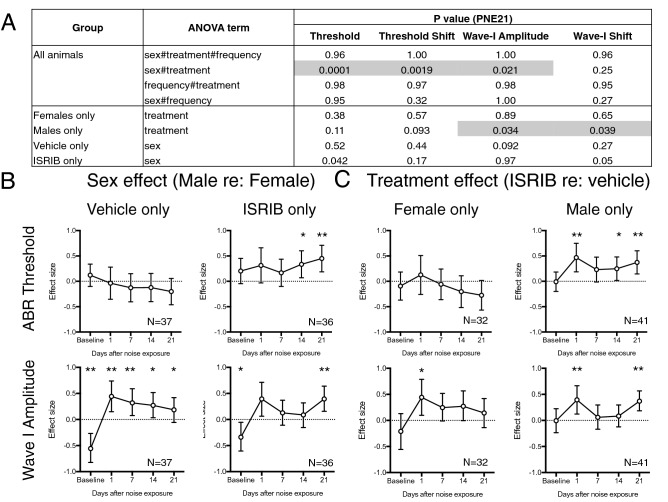
8-week-old female and male wild-type CBA/J mice were treated with a single intraperitoneal dose of 5 mg/kg ISRIB or vehicle 2 h prior to exposure to 2-h, 94.3 or 97.8 dB SPL, 8–16 kHz octave-band noise. ABR was measured at baseline and 1, 7, 14, and 21 days after noise exposure and threshold, threshold shift, wave-I amplitude, and wave-I shift measured at each timepoint. (**A**) 3-way ANOVA revealed significant interaction between sex and treatment at PNE21, so subgroup analysis was performed to evaluate the effect of sex in vehicle- and ISRIB-treated animals separately, and the effect of ISRIB versus vehicle treatment on male and female mice separately. 2-way ANOVA repeated on frequency was used to determine the effect of sex or treatment on noise induced ABR threshold and wave-I amplitude. Statistically significant effects (*p* < 0.05) are indicated by shaded grey boxes. (**B**,**C**). Effect sizes for sex [in either vehicle- or ISRIB-treated animals, (**B**)] or for treatment [in male or female mice, (**C**)] were determined by multiple linear regression for each outcome on sex, treatment, noise exposure level, and frequency. For baseline (Day 0), absolute ABR threshold and wave-I amplitude values were compared; for post-noise-exposure timepoints, ABR threshold shifts and wave-I amplitude shifts (relative to baseline values) were compared. The regression coefficient was divided by the standard deviation for threshold, threshold shift, wave-I amplitude, or wave-I shift to estimate effect sizes. Mean effect sizes ± 95% confidence intervals are shown. *p < 0.05; **p < 0.01 for a significant effect of sex or treatment at each timepoint. Positive effects indicate better hearing (for baseline absolute measurements) or less noise-induced hearing loss (for post-noise-exposure shift measurements) for male relative to female mice (**B**) and for ISRIB-treated relative to vehicle-treated mice (**C**).

We evaluated the effect of drug treatment (ISRIB vs. vehicle) and sex (male vs. female) in this cochlear synaptopathy model across two noise-exposure levels (94.3 and 97.8 dB SPL) and multiple timepoints (0, 1, 7, 14, and 21 days after noise exposure). 8-week-old female and male wild-type CBA/J mice were treated with a single intraperitoneal dose of 5 mg/kg ISRIB or vehicle 2 h prior to exposure to 2-h, 94.3 or 97.8 dB SPL, 8–16 kHz octave-band noise, and effects of sex and treatment evaluated by ANOVA and multiple linear regression (Fig. 3).

Although at baseline, male mice had lower ABR wave-I amplitudes than female mice, with vehicle pre-treatment, noise-induced wave-I amplitude shift was significantly decreased in male mice compared to females across all timepoints, with moderate effect size (Fig. 3B, bottom left). No sex-related differences in baseline ABR threshold or noise-induced threshold shift were noted (Fig. 3B, top left). These findings suggest that even though female mice have higher baseline wave-I amplitudes, they are more sensitive to noise-induced wave-I amplitude reduction. Among ISRIB-treated animals (Fig. 3B, right), noise-induced threshold shifts and wave-I amplitude shifts were both decreased in male compared with female mice at PNE21, suggesting a sex-related late effect of ISRIB pre-treatment.

We next looked at the effect of treatment in female and male mice separately (Fig. 3C). In female mice, ISRIB pre-treatment, compared to vehicle, only had a statistically significant protective effect on noise-induced wave-I amplitude shift at PNE1 that did not persist throughout the experimental timecourse (Fig. 3C, left). In contrast, ISRIB provided statistically-significant reduction in noise-induced threshold elevation and wave-I amplitude reduction at both PNE1 and PNE21 in male mice (Fig. 3C, right). These findings suggest that ISRIB is moderately protective against persistent noise-induced ABR changes in male, but not female mice.

**Figure 4:**
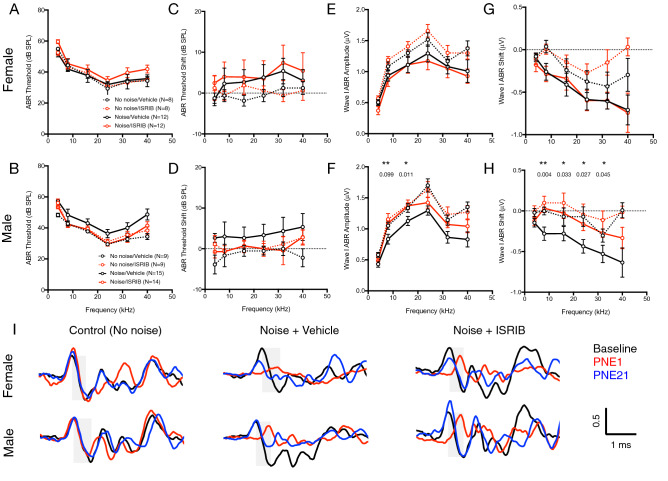
Effect of ISRIB on ABR threshold and suprathreshold wave-I amplitude in noise-induced cochlear synaptopathy. ABR threshold (**A**,**B**), threshold shift (**C**,**D**) and suprathreshold Wave-I amplitude (**E**,**F**) and shift (**G**,**H**) were measured in female (top) and male (bottom) CBA/J mice after a single intraperitoneal dose of ISRIB (red) or vehicle (black) and exposure to 97.8 dB, 8–16 kHz octave-band noise (solid lines), as well in non-noise-exposed control mice (dotted lines). 21 days after noise exposure, there were no significant differences in ABR threshold (**A**,**B**) or threshold shift (**C**,**D**) between mice pretreated with ISRIB (red) or vehicle (black). ISRIB-treated male mice had statistically significant improvement in wave-I amplitude (**F**) and noise-induced wave-I shift (**H**) compared to vehicle-treated male mice. Female mice (**E**,**G**) showed no improvement in wave-I amplitude with ISRIB treatment. Data represent means ± s.e. *p < 0.05; **p < 0.01, with exact p values provided as indicated (Student’s *t* test comparing ISRIB-(red) and vehicle-(black) treated, noise-exposed groups at individual frequencies). (**I**) Representative ABR traces at baseline (black) and post-noise-exposure (PNE) day 1 (PNE1, red) and PNE21 (blue) are shown, demonstrating reduction of wave-I amplitude (peak-to-trough, indicated for baseline reference traces by grey boxes) at PNE1 in noise-exposed mice (red, middle and right). Partial recovery at PNE21 was seen in vehicle-treated mice (blue, middle). Full recovery was seen in an ISRIB-treated male mouse (blue, bottom right), compared with no recovery in an ISRIB-treated female mouse (blue, top right). Control animals (left) did not undergo noise exposure. Traces were high-pass filtered at 200 Hz to eliminate baseline drift and normalized to baseline wave-I amplitude for each mouse (Scale bar: vertical, normalized wave-I amplitude axis; horizontal, time).

These broad effects, revealed through statistical analysis of animals exposed to either 94.3 or 97.8 dB SPL noise, are illustrated by group data on ABR thresholds, threshold shifts, and wave-I amplitudes and shifts in male and female mice exposed to 97.8-dB SPL noise only (Fig. 4). Neither mice treated with ISRIB nor those treated with vehicle exhibited statistically significant threshold shifts upon noise exposure compared to animals that did not undergo noise exposure (*p* = 0.14 for noise exposure by 2-way ANOVA on noise exposure and drug treatment). However, mice did exhibit significant reduction in wave-I amplitude that was mitigated by ISRIB in male mice only (Fig. 4H). Representative ABR traces from male and female mice treated with vehicle or ISRIB after noise exposure, or unexposed, illustrate these findings in individual animals (Fig. 4I). Wave-I amplitude decreased in all noise-exposed animals at PNE1. Vehicle-treated male and female mice had partial recovery of wave-I amplitude at PNE21. Full recovery was seen in an ISRIB-treated male mouse at PNE21, but not an ISRIB-treated female mouse.

### ISRIB protects against noise-induced cochlear synaptopathy

Whereas cochlear synaptopathy manifests physiologically as reduction in the amplitude of wave I of the suprathreshold ABR, loss of IHC-SGN synapses is its definitive anatomic finding. 8-week-old female and male wild-type CBA/J mice were treated with a single intraperitoneal dose of 5 mg/kg ISRIB or vehicle 2 h prior to exposure to 2-h, 97.8 dB SPL, 8–16 kHz octave-band noise. This noise-exposure level was chosen based on the physiology findings reported above, and is consistent with prior studies in similarly aged mice^17^. Temporal bones were harvested 45 days after noise exposure to evaluate long-term synapse counts. We quantified synaptic ribbons between cochlear IHCs and SGNs by identifying colocalization of signal corresponding to antibodies against CtBP2, a pre-synaptic marker, and GluA2, which labels post-synaptic glutamate receptors, in male and female mice.

**Figure 5:**
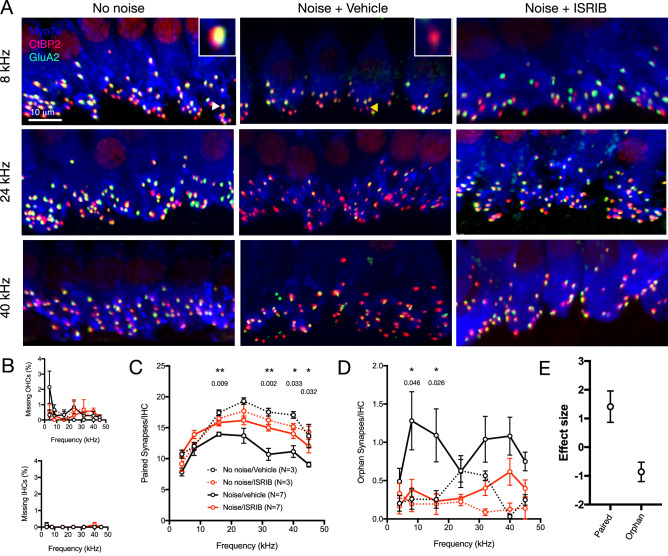
IHC-spiral ganglion neuron synapse count after noise exposure and ISRIB treatment. Female and male CBA/J mice were exposed to 2-h, 97.8 dB 8–16 kHz octave-band noise after treatment with ISRIB or vehicle. Control mice were not exposed to noise. 45 days after noise exposure, cochlear synapses were labelled with CtBP2 and GluA2 and quantified at locations corresponding to the indicated frequencies. (**A**) Representative images from the 8-, 24-, and 40-kHz locations are shown for mice under the indicated conditions. Arrows highlight a representative intact synapse (white; top left, inset) and orphan ribbon (yellow, top middle, inset). Scale bar = 10 μm for all panels. (**B**) Hair-cell counts demonstrated minimal-to-no loss of IHCs or OHCs in any condition [no noise/vehicle: black dotted line; no noise/ISRIB: red dotted line; noise/vehicle: black solid line; noise/ISRIB: red solid line for panels (**B**)–(**D**)]. (**C**,**D**) Number of paired (**C**) or orphan (**D**) synapses in animals pre-treated with ISRIB (red) or vehicle (black) and exposed to noise (solid lines) or unexposed (dotted lines) is shown. Noise exposure led to synapse loss, which was mitigated by ISRIB. Pairwise 2-way ANOVA with repeated measures (on frequency) showed significant effect of treatment on paired (intact) as well as unpaired (orphan) synapses for control (no noise) versus noise/vehicle [*p* = 0.0003 (paired)/0.004 (unpaired)] and noise/vehicle versus noise/ISRIB (*p* = 0.0015/0.009), but not for control (no noise) versus noise/ISRIB (*p* = 0.11/0.23) animals. Data represent means ± s.e. *p < 0.05; *p < 0.01 for Student’s unpaired *t* test at individual frequencies. (**E**) Effect size ± 95% CI for ISRIB treatment (compared to vehicle) on paired and orphan synapses after noise exposure, obtained from the regression coefficient for treatment, adjusting for frequency and sex, and standard deviation of the respective synapse counts.

Consistent with previous reports^1,17^, we observed significant loss of synapses in noise-treated animals (*p* = 0.0003 by two-way ANOVA for noise-exposed vs. unexposed animals), without hair-cell loss (Fig. 5). No difference in synapse counts was found between male and female mice, so they were combined for subsequent analysis. ISRIB protected significantly against noise-induced synapse loss compared to vehicle (*p* = 0.0015, Fig. 5C). No significant difference was seen in synapse numbers between ISRIB-treated mice exposed to noise and control, unexposed mice (*p* = 0.11). Analysis of unpaired, orphan synapses, seen as presence of a presynaptic CtBP2 puncta at inner-hair-cell basolateral membranes with no co-localized post-synaptic glutamate receptor signal, which occur in response to noise trauma^17^, corroborated these results, with noise-exposed, vehicle-treated mice having more orphan synapses compared to unexposed as well as to noise-exposed, ISRIB-treated mice (Fig. 5D). The effect of ISRIB treatment on both paired and orphan synapse numbers, accounting for sex and frequency by multiple linear regression, was large and statistically significant (Fig. 5E).

**Figure 6:**
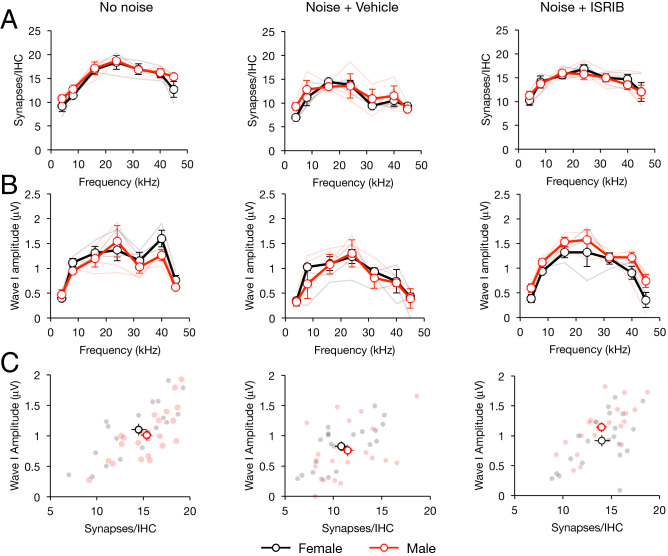
Individual-level comparison of treatment and sex effects on noise-induced changes in synapse counts and wave-I amplitude. Female and male mice were pre-treated with ISRIB or vehicle and exposed to 97.8 dB SPL noise, or unexposed (control; N = 3 in each sex/treatment condition). ABR was performed at PNE21 and synapses quantified at PNE45. (**A**) Comparison between female (black) and male (red) mice reveal no significant sex difference in synapse counts or wave-I amplitudes in control (left), vehicle (middle), or ISRIB (right) conditions. (**B**) Female and male control (left) and noise + vehicle-treated (middle) mice had similar suprathreshold wave-I amplitudes, but noise + ISRIB-treated male mice had significantly higher wave-I amplitudes compared to female mice (right, *p* = 0.0036, 2-way ANOVA for sex and frequency, repeated on frequency). (**C**) Wave-I amplitude and synapse correlation plots are presented. Effect of sex on wave-I amplitude (controlling for paired synapse count) or on paired synapse count (controlling for wave-I amplitude) was analyzed by multiple linear regression, demonstrating no significant differences between male and female mice in control (left) and noise + vehicle (middle) conditions, but significant shift towards higher wave-I amplitude, accounting for synapse number, in male, compared to female, mice under noise + ISRIB conditions (*p* = 0.028). Data represent means ± s.e., with individual animals shown in shaded lines or dots.

Our finding that noise-induced synapse loss was mitigated by ISRIB in male and female mice equally is in contrast with our finding that ISRIB protects male, but not male, mice from noise-induced wave-I amplitude reduction (Figs. 3, 4). Direct comparison and correlation of synapse counts and wave-I amplitudes at all tested cochlear locations/frequencies in ISRIB-treated male and female mice corroborate this finding (Fig. 6). Multiple linear regression demonstrated significantly higher wave-I amplitudes, accounting for synapse counts, in male compared with female mice (*p* = 0.028, Fig. 6C, right).

## Discussion

Our anatomic and physiologic findings demonstrate that ISRIB treatment can reduce cochlear synaptopathy, an emerging phenomenon that may underlie functional auditory disability for millions of individuals. This finding extends our previous work demonstrating the efficacy of ISRIB in preventing permanent noise-induced auditory threshold shifts into the related phenomenon of cochlear synaptopathy, and suggests that a common, targetable mechanism—activation of the UPR—may underlie noise-induced hair-cell degeneration and synapse loss in the cochlea^6^. Additionally, this work expands into sensory systems a large body of research findings in neurological disease models demonstrating the ability to target the UPR to prevent or reverse synaptopathy. Our current finding that ISRIB protects against synaptopathy in the cochlea, therefore, strongly corroborates previous findings in the brain. A link between UPR-mediated neurodegeneration in the brain, hair-cell loss, and cochlear synaptopathy was suggested by recent work in mice deficient for the ER-stress-regulator mesencephalic astrocyte-derived neurotrophic factor (MANF). In this genetic mouse model, ER stress is associated with loss of synapses in the cochlea, accompanied by progressive hair-cell death and hearing loss^18^. The findings that MANF is cytoprotective in neurons and that *Manf* inactivation is associated with loss of synapses in the inner ear suggests that the UPR may act similarly in the brain and auditory sensory cells. Our current findings are consistent with this genetic model. However, our work is the first to provide direct evidence of UPR involvement in an acquired, noise-induced model of cochlear synaptopathy in wild-type mice.

The mechanism by which noise exposure may act through the UPR to cause cochlear synaptopathy is not definitively known, but insight can be obtained through previous work in the central nervous system that may be relevant to the cochlea's response to noise. ER stress, Ca^2+^ release from the ER to the cytoplasm, and UPR activation are well-known in neurons to be involved in glutamate excitotoxicity, a pathophysiologic mechanism strongly implicated in cochlear synaptopathy^19,20^. Our work in genetic and noise-induced mouse models has similarly demonstrated a role for ER Ca^2+^ dysregulation as a link between acoustic overstimulation and UPR activation^6^. Cochlear supporting cells exhibit periodic ER Ca^2+^ release upon acoustic overstimulation^21^, and mice deficient in Tmtc4, a novel deafness gene, exhibit impaired ER Ca^2+^ reuptake, elevated UPR gene expression, and profound post-natal progressive hearing loss^6^. It is possible that noise-induced disruption of ER Ca^2+^ dynamics may affect synaptic function; the ER is the primary store of intracellular Ca^2+^ in presynaptic terminals, and Ca^2+^ is a critical regulator of neurotransmitter release, hair-cell vesicle recruitment, and regulation and timing of vesicle fusion in ribbon synapses^22^. UPR-related modulation of Ca^2+^ homeostasis alters synaptic transmission and plasticity, and ribbon synapse physiology depends on intracellular Ca^2+^ buffering^23^. Inhibitors of ER Ca^2+^ uptake, including thapsigargin, a known UPR inducer, increase resting cytoplasmic Ca^2+^ levels and delay recovery time after depolarization in IHCs^24^. Finally, ryanodine, which stimulates ER Ca^2+^ release, leads to the reversible inhibition of compound action potentials in the auditory nerve, presenting further support of the role of Ca^2+^ in hair-cell synaptic transmission^25^.

In the current study, we also show that the protective effect of ISRIB, as well as the basic phenomenon of noise-induced wave-I amplitude reduction, is highly sex-dependent. Female mice had higher ABR wave-I amplitudes at baseline (Fig. 1), were more susceptible to noise-induced reduction in suprathreshold amplitudes (Figs. 2, 3), and were less responsive to ISRIB treatment compared to male mice (Figs. 3, 4). These findings are similar to published reports in humans, where women exhibit larger baseline wave-I amplitudes despite similar auditory thresholds, as well as greater sensitivity to wave-I amplitude decrease, when compared to men^16,26^. These physiologic sex differences were not reflected, however, in baseline, noise-evoked, or ISRIB-treated synapse anatomy (Figs. 5, 6). The mechanisms underlying this discordance between synapse anatomy and function may include sex-based differences in spatial localization or morphology of synapses, molecular differences such as glutamate receptor expression, or electrophysiologic behavior, and would merit future investigation. More generally, our study highlights the critical importance of including sex as a biological variable in the investigation of treatments for NIHL and cochlear synaptopathy^27^, as well as in the evaluation of UPR modulators such as ISRIB in all disease models.

In conclusion, we have shown that targeting the UPR with ISRIB is an effective means of preventing cochlear synaptopathy in this mouse model, and that with careful and appropriately powered studies, we were able to determine that these effects are sex-dependent. We have previously demonstrated that the UPR is involved in the pathogenesis of permanent NIHL, and that ISRIB can prevent hair-cell death^6^, which is otherwise irreversible. In cochlear synaptopathy, hair cells do not die, but synapses are lost. Unlike hair-cell regeneration, synaptic regeneration in the cochlea does occur in a strain- and species-dependent manner^28^; the ability of ISRIB and other UPR modulators to promote synaptic plasticity and regeneration thus make the UPR an extremely appealing target pathway to treat multiple modes of noise-induced hearing loss—both permanent threshold shift, through prevention of apoptosis and hair-cell death, and cochlear synaptopathy, through prevention of synapse loss and promotion of synaptic regeneration.

## Methods

### Auditory testing and noise exposure

ABR thresholds and growth functions (10–90 dB SPL) were recorded in response to 4–45.2 kHz tone pips in mice at baseline and 1, 7, 14, and 21 days post noise exposure (PNE) as described (RZ6, Tucker-Davis Technologies)^4^. 8-week-old mice were then exposed to 94.3, 97.8, 99.0, 100.7, or 101.4 dB SPL 8–16 kHz octave-band white noise for 120 min in a custom-built, calibrated, reverberant sound chamber^24^. Animals were awake and unrestrained in an acoustically transparent wire cage on a rotating platform throughout noise exposure. Noise exposures were performed at least 24 h after anesthesia for any prior procedures. ABR thresholds were determined as described^4^ and wave-I amplitude measured as the difference between wave-I peak and trough by an investigator blinded to treatment group and confirmed with a custom peak detector (MATLAB). Suprathreshold ABR wave-I amplitude was defined as the average wave-I amplitude in response to stimuli at 70, 80, and 90 dB SPL. To assess the effect of UPR modulation on NIHL, animals were pretreated with intraperitoneal injection of 5 mg/kg ISRIB (MilliporeSigma) or its vehicle (50% DMSO, 50% PEG-400) 2 h prior to noise exposure.

### Experimental rigor and statistical analysis

Wild-type male and female CBA/J mice were obtained from Jackson Laboratories at 6–7 weeks and allowed to acclimatize to our facility for 2–3 days, and baseline ABR measurements completed thereafter by 8 weeks of age. Mice were then randomly assorted into treatment and noise exposure groups for noise exposure at 8 weeks of age. Noise exposures were performed on four mice at a time, one from each treatment group: male and female treated with ISRIB or vehicle. All ABR measurements were performed and scored by an independent investigator blinded to treatment group. Synapse quantification was performed by an investigator blinded to cochlear location/frequency, animal sex, and experimental condition.

Specifics of statistical analysis are described in figure legends. Pairwise comparisons between simple group means were performed with two-tailed, unpaired Student’s *t* test. 2- or 3-way ANOVA was used to assess statistical significance of differences attributable to sex, treatment, noise exposure level, and/or timepoint. When interactions between ANOVA terms were identified, subgroup analysis was performed. Repeated-measures design was used when multiple measurements were made in the same animal that were hypothesized a priori to be affected similarly; for example, wave-I amplitudes at frequencies from 8 to 45 kHz were all expected to be reduced upon noise exposure, and affected by drug treatment in a correlated manner. When there was no a priori assumption of correlated behavior, each measurement was treated independently and correction for multiple comparisons was performed; for example, when evaluating for baseline sex differences in wave-I amplitude across multiple frequencies, each frequency was treated independently and statistical tests corrected for multiple comparisons.

Multiple linear regression was used to quantify the effect of drug treatment and sex on specified outcomes, adjusted for other factors as indicated for each individual test. Effect size was determined by dividing the coefficient of regression for the outcome of interest by the standard deviation of that outcome for a reference group. Effect size was considered as small (0.2), moderate (0.5) or large (0.8). A *p* value of < 0.05 was considered significant. Tests were performed with MATLAB, Stata, and GraphPad.

### Immunohistochemistry and synapse quantification

Synapse quantification was performed by examining co-localization of immunohistochemical staining for CtBP2 and GluA2. Cochleae were harvested 45 days after noise exposure, or in age-matched unexposed controls, perfused with 4% paraformaldehyde, removed from temporal bones, and fixed for 2 h at room temperature. Samples were then decalcified in EDTA at room temperature for 5 days, dissected, and blocked in 5% normal horse serum in PBS with 0.3% Triton X-100 for one hour at room temperature. Primary antibodies were diluted in blocking solution of 1% normal horse serum in PBS with 0.3% Triton X-100 and incubated overnight at 37 °C as follows: mouse (IgG1) anti-CtBP2 (C-terminal Binding Protein) 1:200 (#612044, BD Transduction Labs) targeting pre-synaptic ribbons; mouse (IgG2a) anti-GluA2 (Glutamate receptor subunit A2) at 1:2000 (#MAB397, Millipore) for labeling post-synaptic receptor patches; and rabbit anti-Myosin7a at 1:200 (#25-6790 Proteus Biosciences) for delineating hair cells. Species-specific secondary antibodies were applied for two 60 min incubations at 37 °C as follows: Alexa Fluor 488-conjugated goat anti-mouse (IgG2a) at 1:1000 (#A21131, Life Technologies); Alexa Fluor 568-conjugated goat anti-mouse (IgG1) at 1:1000 (#A21124, Life Technologies); Alexa Fluor 647-conjugated chicken anti-rabbit at 1:200 (#A21443, Life Technologies). Cochlear segments were mounted using Vectashield mounting medium (Vector Labs).

Samples were imaged by confocal microscopy (Nikon Ti). Cochleograms were generated using stitched images at 10 × magnification, and frequencies mapped based on strain-specific data^29^. Missing hair cells were counted from anti-Myosin7 immunostain in segments of 200 μm centered around each frequency and expressed as percentage of missing hair cells. High-powered images were taken at locations corresponding to indicated frequencies using a 60 × oil-immersion objective (1.4 NA) to acquire z-stacks with 0.25 μm step size of the entire IHC synaptic region. Approximately 7–12 IHCs were included in each high-powered view. Image stacks were imported to Imaris processing software to be analyzed in three dimensions. Synapses were then quantified by an investigator blinded to treatment, sex, and cochlear frequency. IHCs were delineated and basolateral cell membrane defined and outlined using anti-Myosin7a cytoplasmic and anti-CtBP2 nuclear stain. Pre-synaptic ribbons (anti-CtBP2 immunoreactivity) and glutamate receptors (anti-GluA2) were visualized at the basolateral pole of each IHC; a colocalized signal was counted as a paired synapse, and CtBP2 signal at the cell membrane in the absence of adjacent GluA2 immunoreactivity was counted as an orphan ribbon. The number of paired and orphan synapses was recorded for each IHC in the high-powered view, and averaged across the individual IHCs in the entire view, to derive the number of synapses/IHC for that cochlear location/frequency. This value (synapses/IHC) was used for all subsequent statistical analyses.

### Study approval

All experimental protocols were approved by the UCSF Institutional Animal Care and Use Committee, and all methods were carried out in accordance with relevant guidelines and regulations.
